# Retraction: Inhibition of anti-viral stress granule formation by coronavirus endoribonuclease nsp15 ensures efficient virus replication

**DOI:** 10.1371/journal.ppat.1014113

**Published:** 2026-04-08

**Authors:** The PLOS Pathogens Editors

Following the publication of this article [1], concerns were raised about Figs 1, 3–6, and S6. Specifically:

- The following panels appear to partially overlap:
  - Fig 3C H1299 Mock G3BP1, DAPI & Merge and Fig 5C Nsp15-H223A G3BP1, DAPI & Merge.
  - Fig 5C PXJ40F G3BP1, DAPI & Merge and Fig 5C Nsp15-H238A G3BP1, DAPI & Merge.
- The following panels appear more similar than would be expected from independent results:
  - Fig 3D Heat shock β-actin and Fig 5D Heat shock β-actin, when the aspect ratio is altered.
  - Fig 5D Heat shock Nsp15 and Fig 5D Sodium arsenite Nsp15.
- When color levels are adjusted to visualize the background, no background signal or noise was detected in the following panels:
  - Fig 1A Mock IBV-N
  - Fig 3C Mock IBV-N (both panels)
  - Figs 4B, 5A, 5C, 6C, 6D PXJ40F Flag
  - Fig S6A Mock PEDV-N

Regarding the concerns about Fig 5D, the corresponding author stated that the published Fig 5D β-actin (Heat shock) panel and one of the Nsp panels are incorrect and provided replacement panels for all proteins (Heat shock and Sodium arsenite) from the original experiment. Editorial assessment of the replacement panels raised further concerns about these data. The corresponding author also provided replacement panels from the original experiment for Figs 3C and 5C.

In addition to the above-mentioned concerns, the corresponding author stated that Figs 7C-E were incorrectly assembled. According to the corresponding author the PXJ40 panels in Figs 7C-E show images of TGEV-nsp15-H226A, SARS-CoV-2-nsp15-H249A, and SARS-CoV-2-nsp15-H249A, respectively.

In light of the above concerns that question the reliability and integrity of these results and the overall data handling, the *PLOS Pathogens* Editors retract this article [1].

XG agreed with the retraction. BG and YL did not agree with the retraction. SF, WW, HW, HC, YS, CM, LT, CS, XQ, WL, MF, and CD either did not respond directly or could not be reached.
